# Quantifying the effect of human practices on *S. cerevisiae* vineyard metapopulation diversity

**DOI:** 10.1038/s41598-020-73279-7

**Published:** 2020-10-01

**Authors:** Marine Börlin, Olivier Claisse, Warren Albertin, Franck Salin, Jean-Luc Legras, Isabelle Masneuf-Pomarede

**Affiliations:** 1 UR Oenologie EA 4577, USC 1366 INRAE, Bordeaux INP, Université de Bordeaux, Bordeaux, France; 2 ENSCBP, Bordeaux INP, 33600 Pessac, France; 3 UMR Biodiversité Gènes et Ecosystèmes, Plateforme Génomique, INRAE, Cestas, France; 4 SPO, Univ Montpellier, INRAE, Montpellier SupAgro, Montpellier, France; 5 Bordeaux Sciences Agro, 33170 Gradignan, France; 6 INRAE, 33140 Villenave d’Ornon, France

**Keywords:** Ecology, Environmental sciences

## Abstract

*Saccharomyces cerevisiae* is the main actor of wine fermentation but at present, still little is known about the factors impacting its distribution in the vineyards. In this study, 23 vineyards and 7 cellars were sampled over 2 consecutive years in the Bordeaux and Bergerac regions. The impact of geography and farming system and the relation between grape and vat populations were evaluated using a collection of 1374 *S. cerevisiae* merlot grape isolates and 289 vat isolates analyzed at 17 microsatellites loci. A very high genetic diversity of *S. cerevisiae* strains was obtained from grape samples, higher in conventional farming system than in organic one. The geographic appellation and the wine estate significantly impact the *S. cerevisiae* population structure, whereas the type of farming system has a weak global effect. When comparing cellar and vineyard populations, we evidenced the tight connection between the two compartments, based on the high proportion of grape isolates (25%) related to the commercial starters used in the cellar and on the estimation of bidirectional geneflows between the vineyard and the cellar compartments.

## Introduction

Vineyards and wineries are ecological habitats that house a community of molds, yeasts and bacteria^1^. The yeast species present on the grape berry are related to a fruit microflora (including mainly *Hanseniaspora* sp.,* Aureobasidium pullulans*,* Pichia* sp.,* Metschnikowia pulcherima*,* Torulaspora delbrueckii*,* Starmerella bacillaris*^2,3^. By contrast, the yeast community in the cellar changes drastically during fermentation with the gradual increase in ethanol and temperature^4,5^, as well as with the use of sulfites for wine making, leading to the domination of *Saccharomyces* sp. *Saccharomyces cerevisiae* has been associated with human fermentations since the dawn of the civilization^6,7^; its diversity is shaped by human activities, especially by winemaking^8–10^. Because of the key role of *S. cerevisiae* in wine production, its genetic diversity has been widely analyzed in the wake of the technological advances in the molecular tools designed to reveal yeast diversity. Since the first exploration of wine *S. cerevisiae* diversity with mtDNA restriction analysis^11^, many more studies have been performed using this technique^12–15^. Almost simultaneously, the polymorphism of the karyotypes of wine yeast revealed by pulsed field electrophoresis has been used as an alternative technique^16–19^. Later inter-*delta* analysis^20–24^ and more recently microsatellite analysis^25–27^ have been used.

Several parameters that could impact the genetic diversity and population structure of wine *S. cerevisiae* have been investigated by different authors. Geographical distance has been the most widely studied environmental parameter, often covering large areas and comparing different regions in a given country. Many species are organized into a metapopulation (i.e., a group of local subpopulations that inhabit discrete habitat patches but interact through dispersal^28^. Knight and Goddard^29^ have shown that the diversity of regional *S. cerevisiae* metapopulations from vineyards were undergoing significant changes between distant areas. These authors have also shown differential migration of this species between regions that may be due in part to the human influence. At vineyard scale level, no spatial differentiation of the *S. cerevisiae* population isolated from spontaneous fermentation is evidenced^30^. Over smaller distances, many vectors may favor the homogenization of diversity such as insects including wasps, bees and fruit flies^31–33^, or migratory birds^34^. Finally, the influence of grape berry varieties on *S. cerevisiae* diversity seems to be low^27,35^.

Vines and cellars are two environments under tight human management, which might impact the global microbial community and influence yeast diversity*.* The use of different phytosanitary products could impact the endogenous yeast populations present on the grape berry^36^. The impact of the organic farming system on the yeast diversity (organic/versus conventional) has not yet been clearly defined till now, with studies reporting controversial results concerning the positive impact or otherwinse of organic farming system on yeast diversity^29,37–40^. Around the wineries, commercial *S. cerevisiae* strains used in the alcoholic fermentation process have been found in grapes samples collected from vineyards within a 200-m radius of the winery buildings^41^. Integrating the endogenous population of the vineyard, the commercial strains would then appear to change the diversity and population structure of *S. cerevisiae*^26,42^. Yet the propagation of commercial starters and their persistence in the environment have been shown to be discontinuous and a non-persistent process^43^.

In addition, few research studies have worked on the possible relationships between the *S. cerevisiae* diversity in the vineyards and in the winery, thus raising the question of the origin of wine yeast^22,23,44^. Strains involved in spontaneous fermentation originated partly from the vineyard and partly from winery^23^. Indeed, a large and diverse yeast population is present mainly in winery surfaces, including *S. cerevisiae* prior to harvesting, and represent a potential reservoir to inoculate the grape must during spontaneous fermentations^45,46^. Characterizing the links between the population from vineyards and that from ferments is thus important from an ecological point of view, but these links are still unclear so far.

The Bordeaux area is one of the world’s most renowned winemaking regions. The first vineyards in Bordeaux were planted in Roman Times, with an expansion during the Middle Ages. The Graves region was the principal wine region, followed by the Entre-Deux-Mers and Saint Emilion^47,48^. These vineyards are planted today with five different red grape varieties: Cabernet Sauvignon, Cabernet Franc, Carmenère, Malbec, Petit Verdot, and Merlot, the latter representing more than 50% of the Bordeaux wine area.

In the present study, 1374 *S. cerevisiae* isolates from 193 samples of Merlot grapes obtained across five regions in the Bordeaux and Bergerac areas, in organic or conventional farming system, and 289 *S. cerevisiae* isolates from 7 cellars were collected. The isolates were genotyped at 17 microsatellite loci. From the data analyses, we show how the human activity associated with the wine making process has shaped *S. cerevisiae* diversity in the vineyards of the Bordeaux region. We also show the significance of the exchange between cellars and vineyards populations.

## Results

### Yeast collection from grapes

From the organic (n = 13) and conventional (n = 14) wine estates, we collected 193 samples of grapes (134 in 2012 and 59 in 2013) among which 166 (107 in 2012 and 59 in 2013) initiated a fermentation (Table 1; Supplementary Fig. S1). From those for which the production of CO_2_ indicated that more than the half of the glucose and fructose of the must had been fermented (Supplementary Fig. S1), we collected 3369 colonies including of 1374 *S. cerevisiae* isolates, and genotyped 1002 individuals (Table 1 and Supplementary Table S2).

**Table 1:** Summary of grape samples collected in Bordeaux and Bergerac regions during two consecutive years, number of fermentations giving *S. cerevisiae*, number of *S. cerevisiae* isolates and unique profiles (Nb: number).

Appellation	Vintage	Farming system	Nb. of wine estate sampled	Nb. grapes samples	Nb of sample with fermentation	Nb. of fermentation giving S.c isolates	Mb. of S.C isolates	Nb of *S.c* unique profiles
Bergerac	2012	Organic	1	5	5	3	57	9
	2013	Organic	1	5	5	2	16	6
Medoc	2012	Organic	2	15	10	3	43	19
	2013	Organic	1	6	6	2	34	12
	2012	Conventional	3	11	8	3	77	31
	2013	Conventional	1	3	3	2	25	13
Pessac-Leognan	2012	Organic	3	17	17	8	131	61
	2013	Organic	1	5	5	0	0	0
	2012	Conventional	5	31	25	12	165	76
	2013	Conventional	4	20	20	13	198	81
Entre Deux-Mers	2012	Organic	1	5	5	1	2	2
	2012	Conventional	1	5	3	2	19	5
Saint Emilion	2012	Organic	4	21	17	3	21	4
	2013	Organic	2	10	10	1	19	6
	2012	Conventional	5	24	17	7	87	27
	2013	Conventional	2	10	10	6	108	50
Total				193	166	68	1002	402

In order to evaluate the global *S. cerevisiae* diversity in the different vineyards, four indices were calculated: the Shannon (H′), the Simpson index (D), its opposite (1 − D) and the Pielou evenness index (Table 2). Although the number of sampling sites between organic and conventional wine estates was similar (13 and 14, respectively), the number of *S. cerevisiae* grapes isolates was approximately twice as high in conventional farming: 662 grapes isolates, compare to organic: 340 grapes isolates from organic operations (Table 2a). This led to higher values of the 3 diversity indices for *S. cerevisiae* in conventional farming when compared with organic farming. The gap between the 2 Simpson’s indices of diversity (1 − D), which gives more weight to common or dominant species, was smaller, indicating that the diversity of common and dominant isolates was alike. This is confirmed by the comparison of rarefaction curves for possible population diversity in both farming systems (Supplementary Fig. S2) since 6169 and 3620 strains were inferred over 1000 samples, for conventional and organic farming respectively. At the scale of the region, without taking into account the farming system, we obtained higher diversity indices for Medoc, Saint Emilion and Pessac-Leognan than for Bergerac and Entre Deux-Mers (Table 2b) with similar genotypes abundance and diversity (equitability index and 2 Simpson’s indices) for the first three appellations and lower indices for the last two appellations. Despites our efforts, this sampling strategy did not provide enough unique profiles to exhaust the diversity of the whole region, as shown in the rarefaction analyses which estimated that these genotypes at the entire regional scale were sampled from an underlying population containing 6777 different genotypes (with 95% confidence limits of 3194).

**Table 2:** Diversity of *S. cerevisiae* isolates under organic and conventional farming.

	Organic farming	Conventional farming			
**(a)**					
Number of individuals	340	662			
H′ (Shannon Index)	4.09	4.86			
J′ (Equitability Index)	0.68	0.80			
1/D (Simpson index)	32.47	66.83			
1 − D (Simpson complement)	0.97	0.99			

	Medoc	Pessac Leognan	Saint Emilion	Bergerac	Entre Deux-Mers
**(b)**					
Number of individuals	149	524	235	73	21
H′ (Shannon Index)	3.78	4.69	3.73	1.9	1.22
J′ (Equitability Index)	0.62	0.77	0.62	0.31	0.20
1/D (Simpson index)	24.26	56.17	22.51	4.49	2.15
1 − D (Simpson complement)	0.96	0.98	0.96	0.78	0.53

Shannon index (H′), equitability index (J′) and Simpson index (1/D) and its complement index (1 − D). Analyses of the 1374 *S. cerevisiae* obtained after microsatellites analyzes depending on (a) the type of farming system, organic or conventional and (b) the different appellations of Bordeaux and Bergerac.

### *Saccharomyces cerevisiae* strains diversity isolated from grape and vat samples

**Figure 1:**
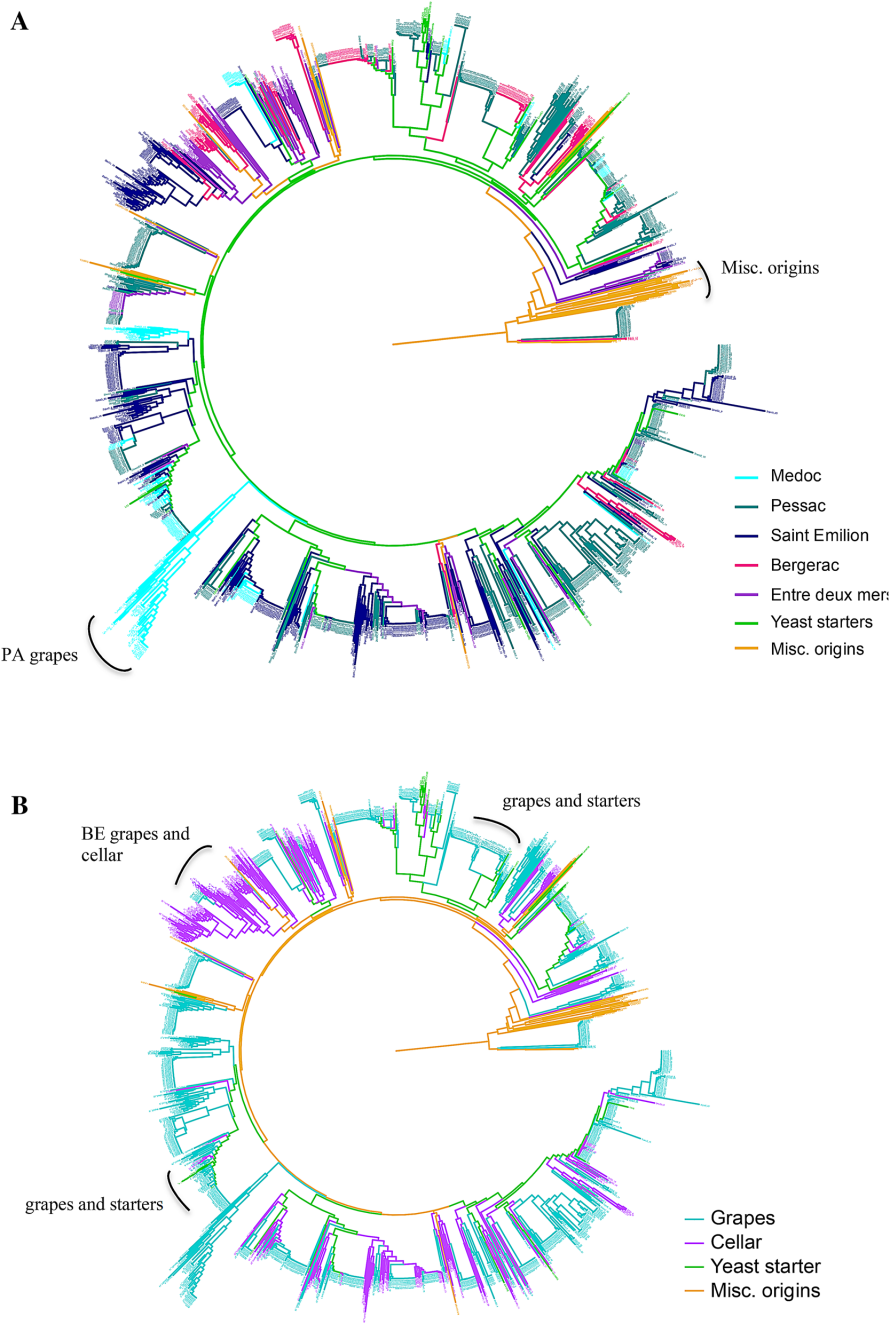
(**A**,**B**) Genetic diversity of *S. cerevisiae* grape (1002) and cellar (289) isolates from the Bordeaux and Bergerac region in comparison to 33 commercial strains (yeast starters, green) and 33 strains of various origins (Misc. origins, orange). The neighbor joining tree was built from a Bruvo’s distance matrix based on the polymorphism at 17 loci followed by Neighbor joining tree clustering and rooted at midpoint. (**A**) Colors are according to the Appellation, whetherisolates are from grapes or cellars, (**B**) colors are according to the compartiment of origins (blue grapes, and pink cellars).

The complete dataset of 1002 grape and 289 vat isolates was compared to 33 commercials yeast starters and 35 strains isolated from various substrates^49^ (Supplementary Tables S2 and S3) from a tree constructed with the Bruvo distance^50^ (Fig. 1A,B). This neighbor-joining tree revealed a cluster with strains from non-wine origins (Misc.origins), while wine strains whose genome had been sequenced were found to be mixed with Bordeaux grape strains (Fig. 1A). Some clinical and soil isolates were clustered among wine isolates in agreement with their genomic characterization^49^. Many clusters gathered identical grapes isolates indicating clonal expansion. With some exceptions, it should be noted that several grapes isolates clustered according to the wine estate where they had been isolated, along with one cluster (“Pa” grapes, Fig. 1A) stood out by the atypical lengths of their branches. Grape strains were also clustered according to their appellation, including different wine estates (Fig. 1A). Last some clusters gathered grape and vat isolates from the same wine estate (see “be” Fig. 1B) whereas other clusters contained grape isolates closely related to commercial starters (Grapes and starters, Fig. 1B).

### Population structure of grape isolates

**Figure 2:**
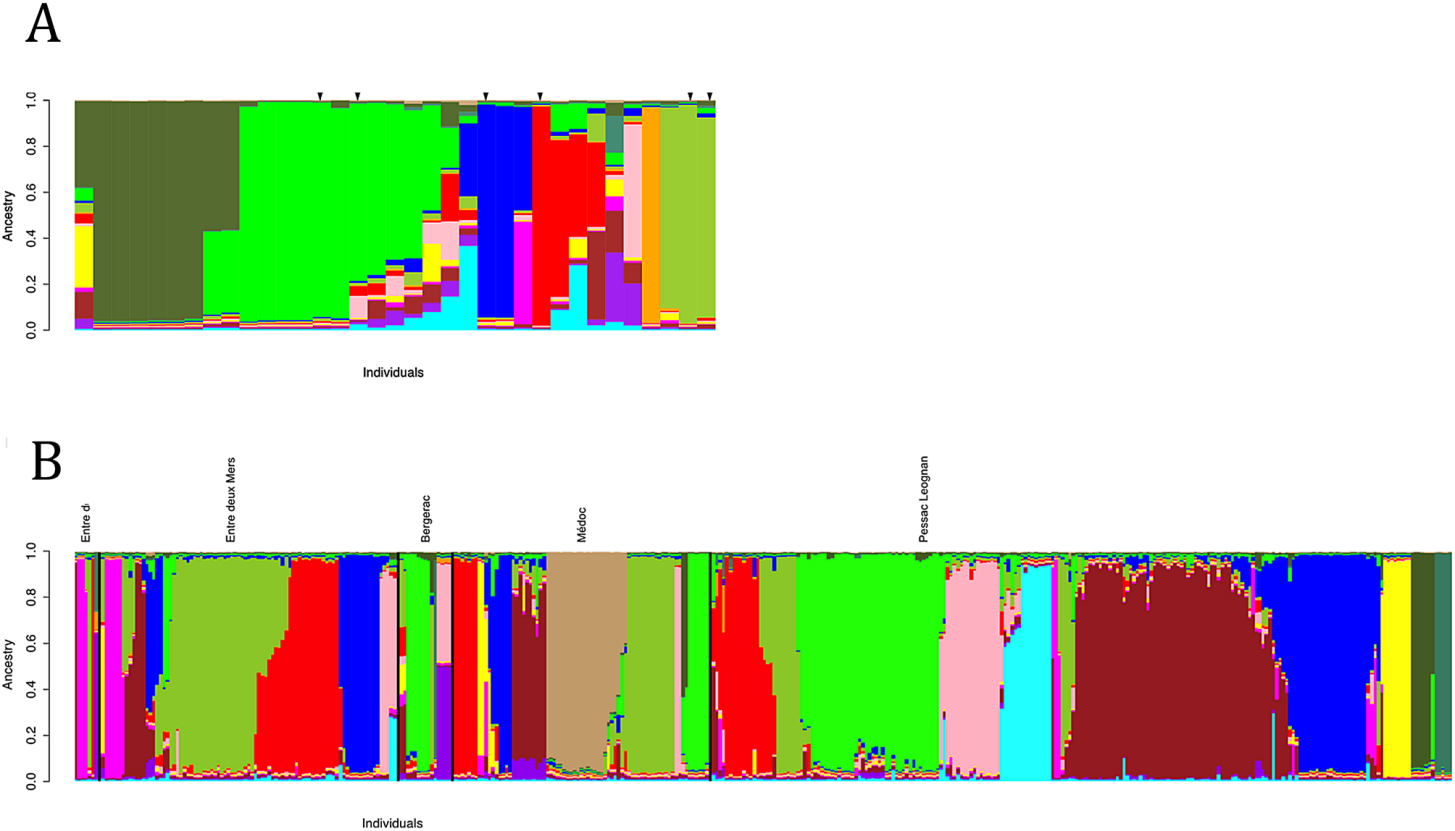
Inference of population ancestry using InStruct (optimal K = 14). Analyses were performed on a dataset containing 402 grape strains and 33 commercial strains a. Barplot presenting the ancestry of the 33 commercial strains b. Barplot presenting the ancestry of the grapes isolates.

After the removal of identical clones detected on the same wine estate, the dataset contained 402 grape isolates, corresponding to 398 unique genotype profiles. The ancestry profiles of these individuals, including commercial strains, were then further inferred from the microsatellite dataset using the Bayesian clustering method implemented in InStruct^51^. Given the microsatellite data set, the optimal number of ancestral populations inferred was K = 14 and the percentage of ancestry identified for yeast starters or grapes strains is presented in Fig. 2a,b, respectively. The Pessac Léognan and Medoc appellations appear also to have a small proportion of specific and unique ancestral population linked with one wine estate, whereas commercial yeast starters presented a global ancestry similar to the grape isolates.

### Relation between grape isolates and industrials yeast starters

**Figure 3:**
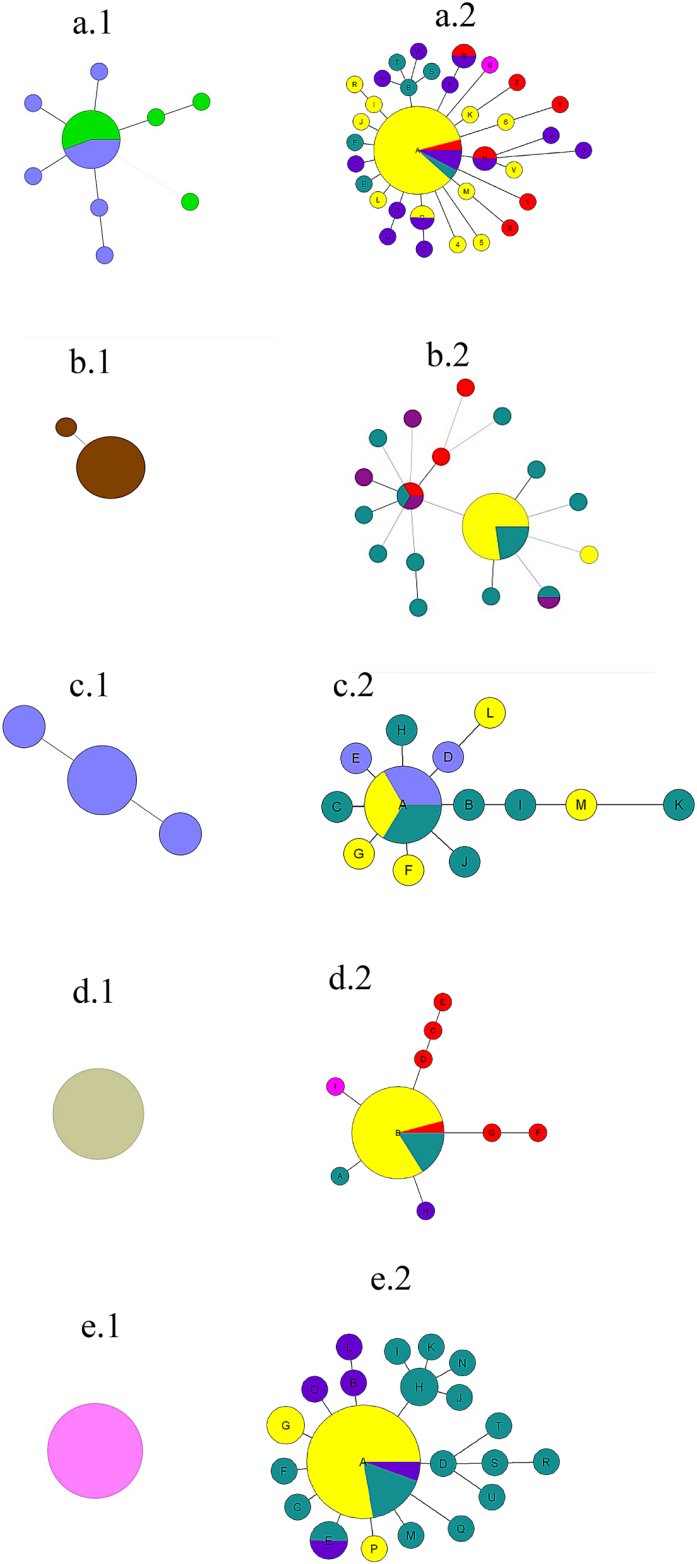
Comparison of the spanning trees presenting the relation of grape isolates with their related yeast starters, to the spanning trees including clones isolated from of a yeast starter production. Left-hand graphs numbered with 1 present the diversity found among strains from industrial preparation. Right-hand graphs numbered with 2 present the association grapes isolates and the corresponding commercial starter. Color code (1) (**a**) 522D/F33 (green and light blue); (**b**) FX10; (**c**) X5; (**d.1**) F15; (**e.1**) VL1. Color code (2): commercial strains, yellow; Médoc, purple; Saint Emilion, red; Entre Deux-Mers, fuchsia; Pessac-Léognan, green; Bergerac, dark blue.

The dendrogram highlighted clusters gathering starters and grapes isolates. As the presence of yeast starters in the vineyards has previously been reported at variable frequencies^41^, we searched for the isolates strongly related to the most representative commercial yeast starters used in the Bordeaux area, (522D/F33, FX10, F15 used for red wine fermentation, and X5, VL1 used for white wine fermentation). The relationships of 100 isolates sharing at least 75% of their alleles with one commercial yeast starter are presented in 5 spanning trees (Fig. 3). For each yeast starter, 4–5 grapes isolates had exactly the same microsatellite profile; in addition, 2–16 others had allelic differences at one or two loci, suggesting clonal variants (Fig. 3a2/b2/c2/e2). All grape isolates in the group related to the commercial strain F15 presented more than two alleles different from the commercial strain (Fig. 3d2). Yeast starter VL1 selected in 1987 from the Bordeaux appellation clustered with numerous grapes isolates from Pessac Léognan, the main white wine production area in Bordeaux (Fig. 3e2). In addition, we genotyped 10 clones isolated from different industrial batch productions of each starter in order to evaluate their genetic homogeneity. Spanning trees based on microsatellite profiles of clones and original isolates of each commercial strain (Fig. 3) indicated slight differences according to the yeast starter. For F15 and VL1, all starter isolates gave identical profiles to their respective original strains (Fig. 3d1,e1), while for X5 two isolates presented differences at one locus (Fig. 3c1). The two last starters 522D and F33 presented the same microsatellite profile, but 5 out of 20 and 3 out of 20 isolates presented variations of F33 and 522D, respectively, thus creating 8 additional profiles to the main one with one or two divergent alleles for each (Fig. 3a1). Identical karyotype profiles were obtained for the commercial strains FX10, X5, F15 and VL1 and their respective vineyard clonal variant based on microsatellites patterns whereas different karyotypes were obtained for vineyard isolates for the 522/F33 genetic background, but similar ones to clonal variants isolated from different industrial batches. All in all, the microsatellite and karyotype analyses confirmed the close genetic relationships between the industrial starters and grapes isolates (Supplementary Fig. S3 and Supplementary Table S7).

As VL1 was isolated 30 years ago from the local indigenous population, the question is raised as to whether the isolation of strains related to VL1 on grapes derives from its presence as a historically highly frequent genotype or from its recent use. The first hypothesis should provide a cluster of local closely related strains with several differences between them and in quite high frequencies whereas the second hypothesis should provide a network of genotypes centered around the starter with many branches containing a single mutation, characteristic of a recently expanding population. The different spanning trees obtained here are always in agreement with the second hypothesis, which suggests that the isolates derive from the yeast starters. The higher genetic variability observed on these grape isolates in comparison to the starter suggests that these variations did not occur during the industrial-scale multiplication of the starter, but more likely result from the multiplication in the vineyard/cellar environment over a longer period, with their spread into the cellar and the vineyard.

We evaluated whether the distance between the sampling site and the winery could explain the frequency of *S. cerevisiae* encountered in grapes samples fermentation, as well as the frequency of strains related to commercial starters. These analyses were done only on the 2012 harvest season. The distances between grape sampling sites and the closest cellars varied between 28 and 380 m. At this scale, no relation could be seen between the number of grapes samples containing *S. cerevisiae* for each wine estate and distance (Supplementary Fig. S4a) nor between the percentage of grape isolates related to commercial strains and distance (Supplementary Fig. S4b).

### Impact of geography and farming system on population structure of *S. cerevisiae* in the vineyards

Because the fraction of yeast starters used in the different regions may lead to a spurious increase in similarities between the regions, we removed all strains with profiles presenting more than 75% identity with industrial strains. This reduced the data set from 402 to 302 grape isolates, meaning that 1/4 of the grape isolates collected were closely related to commercial strains. Basic information on multilocus genotype per samples and heterozygosity are given as supplementary information (Supplementary Table S6). When analyzing the heterozygosity of the different sampled populations, we could observed for all of them a deficit in heterozygosity for all populations, very likely resulting from the *S. cerevisiae* life style, but two estates presented populations with a more pronounced deficit in observed heterozygosity (“pa” and “lh”), despite a similar allelic richness.

### Testing for geographic differentiation

In order to evaluate the potential influence of appellation, wine estate or farming as potential factors on population structure, we performed an AMOVA (Table 3a).

**Table 3:** Quantifying the impact of region, estate and vine management system on the population structure of *S. cerevisiae* in the vineyards.

Model tested for AMOVA	Variation source	df	Phi for the factor	Phi for individuals	P value
**(a)**					
Appellation	Appellation	4	0.0902	0.6908	0
Appellation/estate	Appellation	4	0.0237	0.6851	0.044
	Estate	14	0.1800	0.6851	0.999
Estate	Estate	18	0.2010	0.6857	0
Appellation	Appellation	4	0.0902	0.6908	0
farming system	farming system	1	0.0351	0.6932	0
Appellation/vine management	Appellation	4	0.0083	0.6899	0.5974
	Vine Management	4	0.1369	0.6899	0.979

	Médoc	Pessac Léognan	Saint Emilion	Bergerac	Entre-deux-Mers
**(b)**					
Number of individuals	51	164	71	11	5
Médoc	0	0.001	0.001	0.001	0.001
Pessac Léognan	0.104	0	0.001	0.001	0.001
Saint Emilion	0.127	0.058	0	0.001	0.001
Bergerac	0.151	0.089	0.139	0	0.001
Entre-Deux-Mers	0.172	0.134	0.120	0.163	0

(a) Impact of region, estate and vine management system on *S. cerevisiae* diversity in vineyards of the Bordeaux and Bergerac regions. (b) Pairwise Fst values between appellations of Bordeaux and Bergerac regions after removing all grape strains associated with commercial wine strains. The values are compared to the distribution obtained by randomization, and the estimated P value is given in the upper half of the matrix.

The appellation and the wine estate have a highly significant effect when considered solely. However, when those factors are combined, only the impact of “appellation” on population structure remains significant whereas the “estate” factor does not (Table 3a). This may likely come from differences in the contribution of each estate to the global variance in each appellation sampling. Indeed a DAPC performed on the genotype dataset points to the high contribution of some estates to the global variations such as “pa” or “bc” (Supplementary Fig. S5).

The pairwise Fst distance matrix is a complementary way to display population differentiation (Table 3b). The Fst values between appellation indicating significant low to moderate differentiations (0.058–0.172). Last, we used the ancestry profile inferred with InStruct, and we evaluated with ObStruct^52^ we evaluated whether the correlation of the sampled populations with their ancestry profile explains their differentiation (Supplementary Table S8). Differentiations were stronger for three populations, the Médoc, Saint Emilion and Bergerac appellations which can be visualized from a canonical discriminant analysis performed on ancestry (Supplementary Fig. S6). More divergence can be observed for the two first appellations, which present the highest contribution to population structure (Supplementary Table S9). As observed with the DAPC, one wine estate contributed predominantly to the metapopulation for the Médoc appellation and one for Pessac Léognan.

This differentiation in the population structure might also result from a spatial pattern of genetic variation, but with a Mantel test (which relies on a randomization procedure), no significant correlation was observed between the Fst matrix distance and the geographic distance. We alternatively inferred gene flow between the different appellations, using a Bayesian coalescent approach implemented in the Migrate software, and using the method proposed by Sundqvist et al.^53^. Both methods indicate asymmetric migrations, and St Emilion as a source of migrants towards Medoc and Pessac Léognan (Supplementary Table S10, Supplementary Fig. S7).

### Testing for the impact of the farming system

The AMOVA revealed an impact of the farming system on the population structure (Table 3a). However, when combining appellation and farming system, neither of the two factors appeared as significant. When comparing the two farming systems, the estimation of the differentiation between the two metapopulation from the Phi_St statistics (0.035) and from the Fst distance statistics (0.036) showed a low but significant differentiation (P value < 0.001 inferred from a randomization test), indicating that despite an apparently lower impact on the occurrence recovery of *S. cerevisiae*, organic farming system have little impact on *S. cerevisiae* diversity.

### Relation between grape and cellar *S. cerevisiae* diversity

Grapes are one source of the *S. cerevisiae* strains involved in the winemaking process, but the link between grape and cellar *S. cerevisiae* populations is still poorly characterized till now. Thus, we aimed to evaluate connections between grape and cellar metapopulations.

We collected and genotyped 289 *S. cerevisiae* strains from 11 spontaneously fermenting musts in 7 cellars. Clusters gathering grape and vat isolates were highlighted in the dendrogram tree (Fig. 1B). After filtering for clonality and after removing strains related to with commercial yeast starters, this data set decreased to 225 cellar-associated unique *S. cerevisiae* profiles.

In a given appellation, the genetic differentiation between the grape and cellar population is significant with moderate to high Fst values between 0.09 and 0.22 (Supplementary Table S11a). In contrast, the comparison of these cellar populations to that of the vineyards at the entire Aquitaine region level (Supplementary Table S11b) indicated a low differentiation except for two estates (“cos” and “bc”). Because this picture may have resulted from clonal amplification in the vats, we built a balanced subset of strains. We chose 5 cellars and neighboring vineyards and sampled down to 20 individuals per domain. This resulted in a much lower differentiation between cellars and grapes metapopulations: Fst = 0.03 ± 0.001 (mean if 100 random sub-samples).

As this low population differentiation may result from unbalanced geneflows between the grapes and the cellar, we inferred this genetic exchange between the cellar and the grapes using the Bayesian coalescent approach implemented in MIGRATE. A similar theoretical population size was inferred for the grapes and cellar metapopulations, which were found to be connected by geneflow, higher from the grape to cellar metapopulations than from the cellar to the grapes, indicating that both compartments are tightly connected (Table 4). A similar estimation performed with the method proposed by Sundqvist et al.^53^ confirmed the bidirection migration between the two compartments, as no significant asymmetry in gene-flow was observed.

**Table 4:** Estimations of geneflows between cellar and grape metapopulations inferred with Migrate.

Parameter	Mode	95% confidence interval
Theta_grapes	5.69	[4.28–7.4]
Theta_cellar	3.78	[2.99–4.49]
Migration rate cellar—> grapes	55	[25–83]
Migration rate grapes—> cellar	191	[166–226]

Bayesian confidence intervals are obtained from the posterior distribution of the parameters.

## Discussion

In this study, the diversity and population structure of *S. cerevisiae* were analyzed in the Bordeaux and Bergerac region. The only study describing the diversity of *S. cerevisiae* associated with grapes in the Bordeaux region was conducted in 1992 by Frezier, and relied on karyotype analysis. It reported that a small number of strains were dominant during non-inoculated alcoholic fermentation, irrespective of the variety or the time of harvest considered^17^. However, given the low resolution offered by pulsed gel electrophoresis, a fine scale analysis of the yeast population structure could not be achieved. In this study, we aimed to conduct an in-depth genetic diversity analysis of the *S. cerevisiae* population structure, based on the robustness of microsatellite markers with a higher number of loci (17 loci). Because it was performed on a large scale, with five wine-producing appellations, 25 wine estates including 2 farming systems over two consecutive years, this study is unique compared to other studies which only considered 2 wine estates for each farming system^39,43^.

Our Merlot grape variety sampling served to estimate that the Bordeaux and Bergerac region is expected to contain a much wider diversity of *S. cerevisiae* strains, with more than 6000 unique genotypes. This region scale estimate is nearly four time higher than the estimate of 1700 inferred for the metapopulation sample of New Zealand vineyard^29^, which may be related to the recent arrival of the New Zealand wine yeast population from European wine yeasts^54^.

The principal goal of this study was to evaluate the factors that may explain vineyard-associated *S. cerevisiae* diversity and population structure: the geographic factor associated with the “appellation”, the wine-estate and organic versus conventional farming systems. The influence of pest management systems on vineyard-associated yeast biodiversity is a key issue for the wine industry in the context of sustainable agriculture but is still a controversial ecological topic. Some authors showed that the use of phytosanitary treatments in the vineyards could negatively impact the yeast population diversity^55,56^, especially that of *S. cerevisiae* yeast^38^. But other studies have reported higher *S. cerevisiae* strains diversity in conventional must fermentation in comparison to organic ones and have demonstrated that fungicides have no impact on yeast counts on grapes and during the alcoholic fermentation^37,39^. In our study, based on a large numbers of wine estates^25^, the global estimation of the number of genotypes from a rarefaction curve, indicates an approximately two folds increase in the number of *S. cerevisiae* grapes isolated from grapes in vineyards under a conventional farming system when compared to organic operations. Similar results were obtained recently from Spanish vineyards showing intermediate to low *S. cerevisiae* strain diversity for organic vineyards but higher levels for conventional practices^40^. A lower fungal diversity of the microbial community due to repeated fungicide applications in conventional farming could explain this higher diversity index for *S. cerevisiae* observed among conventional farming systems. We can hypothesize that a lower competition for nutrients could offer more ecological space to *S. cerevisiae*. In addition, a low differentiation was observed between the two farming systems which indicates that this factor is not a main driver of the *S. cerevisiae* population structure in the Bordeaux and Bergerac area.

The persistence of commercial *S. cerevisiae* starters in the vineyard and its impact on autochthonous yeast diversity is another topic that has been investigated by several authors in different wine producing areas. Previous studies reported concordant results indicating an infrequent dissemination of commercial yeast in the vineyard surrounding the winery, and show that the dissemination is restricted to short distances (maximum distance of 100 m around the dissemination area and in a limited period of time)^41,43^. By comparing indigenous *S. cerevisiae* genotypes with a database of 79 commercial wine strains commonly used by the wine industry, Gayevskiy showed that only a few isolates shared one microsatellite allele with commercial starters, thus supporting the concept that a diverse natural population resides in New Zealand^35^. Our data including 23 vineyards and 7 cellars reported around 25% strains isolated from grapes with a close genetic relationship with the commercial starters, echoing those of Viel et al.^57^ in Italy. The distance separating the closest cellars and the sampling area ranged from 1 to 350 m, supporting the fact that commercial strains can be transferred to the vineyard at longer distances than previously reported, mixing with the endogenous grape strains population. Dispersal of commercial strains could be mediated by water run-off, macerated grape skins at dumping sites^41^, but also by drosophila^33^, or even by the air through CO_2_-extraction systems^58^. The clonal variations observed among grape isolates related to industrial starters could be an indication of a long-term dissemination of yeast starters in the environment. This hypothesis is reinforced by the fact that the use of industrial yeast to inoculate grape juice has been widespread in the Bordeaux wine producing area for over 40 years. Even though some of these starters have been isolated from the Bordeaux region (e.g. VL1 in 1987), the high diversity estimated from our sampling and the spanning trees centered on yeast starters make it likely that these clusters are derived from the starters and not from the local clones from which starters are derived. Cellars could contribute to the vineyard diversity enrichment by enologically relevant *S. cerevisiae* strains that were previously selected for their fermentative properties. However, the transition from nutrient-rich musts to nutritionally scarce natural environments has been shown to induce adaptive responses for the clonal variants that have diminished capacities related to winemaking in comparison with the reference strain^42^. It will be interesting to extend this study at the genomic and phenotypic level to the biological material provided by this work.

The question of regional differentiation is still open for winemakers and wine microbiologists. It has been shown that within regions (encompassing a radius of 100 km) in New Zealand, there is no compelling evidence of genetic differentiation between managed niches and native ecosystem and within managed ecosystems^29^. However, regional delineations of natural *S. cerevisiae* populations have been evidenced^29,35^. In this study, we aimed to test for geographic differences in *S. cerevisiae* populations at the appellation scale in the Bordeaux and Bergerac wine-producing region. A population-based analysis revealed differentiation between appellations, indicating a certain population structure. This pattern was not explained by geographic distance. Insects like bees, wasps and fruits flies, or even birds, could disseminate *S. cerevisiae* especially when the different regions are apart less than 100 km apart^31,32,34^ and could thus be responsible for the homogenization of *S. cerevisiae* within regions^29^. In the case of the Bordeaux wine-producing area, our results suggest higher migrations between Pessac Léognan, and Saint-Emilion which are consistent with low pairwise Fst between these two appellations. When comparing the net flux for each region, both Pessac-Léognan and Saint-Emilion appear to export migrants, whereas the Medoc appear to be a region importing strains from other appellations.

A critical feature of the relevance of yeast diversity for winemakers is the correspondence between cellar population and wine estates grape populations. Using a balanced sample, we show for the first time a low differentiation between cellars and grapes populations for the first time. We were able to show that the connectivity between the two groups arises from the flow of grape strains from cellar to vineyard, illustrated by the high frequency of related commercial related strains isolated from grape samples. This is a new indication of a long-term dissemination of yeast starters in the environment. The estimates of the internal and external migrations between cellars and vineyards attest to the importance of the flux of yeast cells from the vineyard entering the cellar, which has been suggested but never been properly estimated. The small differentiation also suggests few differences in the life styles between the cellars and the vineyards.

Overall, this study provides original results on the diversity and population structure of *S. cerevisiae* within an historical wine making region. The geographic appellation and the wine estate significantly impact the *S. cerevisiae* population structure, whereas the type of farming system has a weak global effect. Our results do not give credits to the concept of clones isolated at high frequency and specific to a given appellation (so called “terroir strains”). However, at the appellation scale, the populations presented some structure suggesting the presence of region-specific populations. At a smaller scale, some wine estates presented specific populations, but their persistence should be further evaluated in the long-term.

One main feature is the high inter-connection between vineyard and cellar population, making it an almost continuous ecosystem that does not have a single direction, from grapes to cellars, but also from cellars to grapes.

## Materials and methods

### Samples collection and processing

**Figure 4:**
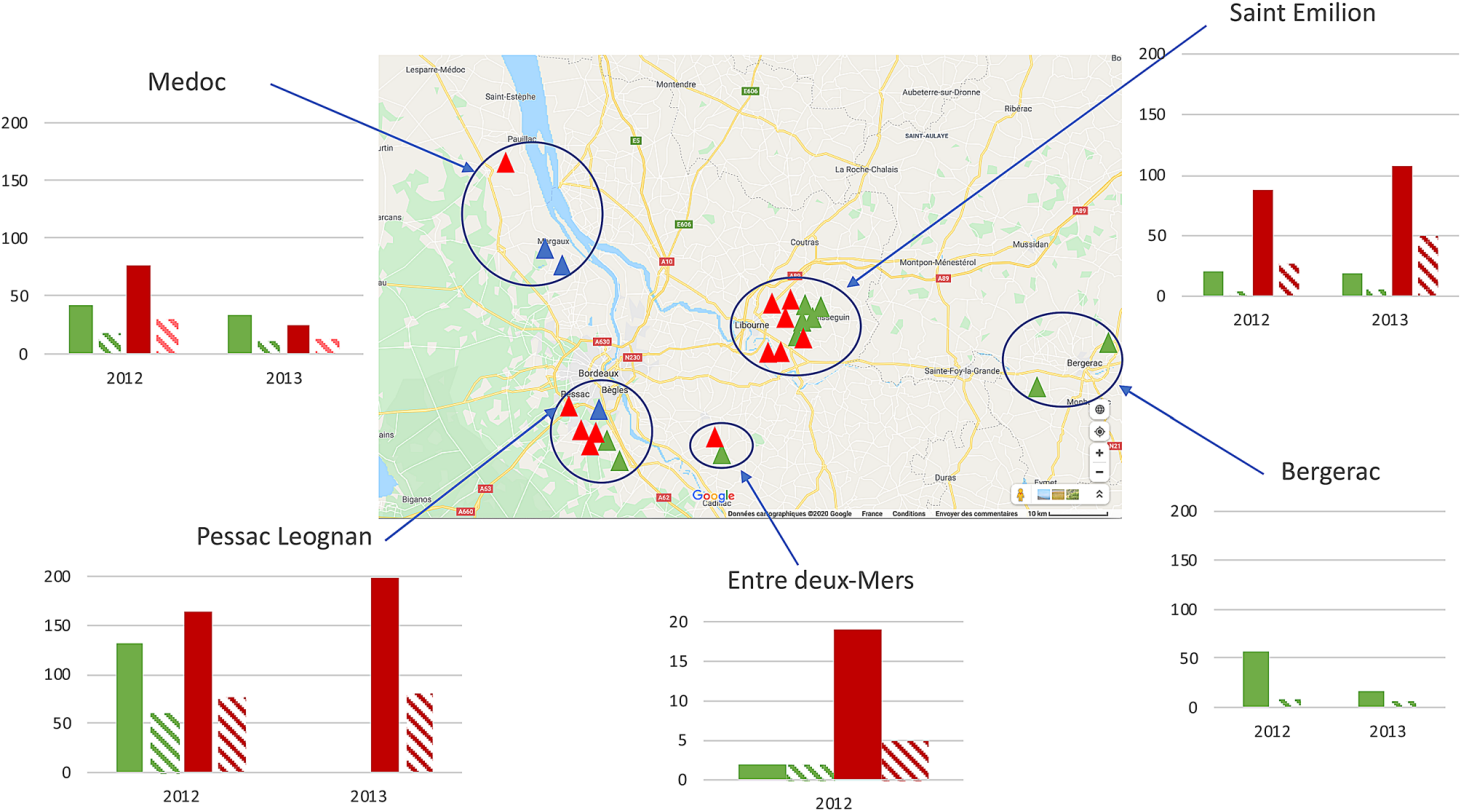
Geographic localization of the wine estates in the appellations of the Bordeaux and Bergerac regions. Green labels represent vineyards with an organic farming system, red with a conventional one and blue, vineyards managed with both an organic and a conventional farming systems. For each Appellation, a summary of the number of *S. cerevisiae* isolates (green: organic; red: conventional) and unique *S. cerevisiae* genotypes (hatched green: organic; hatched red: conventional) for 2012 and 2013 is given.

Five wine producing areas in the Nouvelle-Aquitaine region of the south west of France were selected corresponding to 4 Bordeaux appellations: Medoc, Pessac Leognan, Entre-Deux-Mers, Saint Emilion and to one in Bergerac (Fig. 4). In total, 24 wine estates were sampled, 9 with an organic farming system, 12 with a conventional farming system and 3 with both in organic and conventional farming systems (Fig. 4, Table 1, Supplementary Tables S1 and S2). Two wine estates were sampled in Bergerac and Entre deux-Mers, 3 in Medoc, 7 in Pessac-Leognan and 10 in Saint-Emilion. For each wine estate, between 5 and 12 samples of 2 kg of healthy and mostly undamaged Merlot grapes were collected few days before the harvest across two consecutive vintages thus resulting in 193 grape samples. In 2012, 23 wine estates were sampled, 11 conducted in organic (63 grape samples) and 12 in conventional farming system (71 grape samples), and in 2013, 6 organic (16 grape samples) and 6 conventional wine estates (33 grape samples) were selected (Table 1). In addition, fermenting vats from 7 organic wineries were sampled from the vats in cellars for must at 75% of the fermentation (Supplementary Table S2). In 2012, 6 wineries were sampled, of which 5 for grapes, and in 2013, 3 wineries were sampled of which 2 were also sampled for grapes (Supplementary Table S2). Sampling was not relevant for the other wineries associated with vineyard sampling as they used yeast starters.

### Fermentation and strains isolation

Yeast strains were isolated from the juice extracted from the grapes after enrichment to ensure the presence of *Saccharomyces* strains. Briefly, for each of the 193 fruit samples, the grapes were crushed, then macerated for 2 h with their skins and seeds. After addition of 50 mg/l of SO_2_, the extracted juice was fermented at 21 °C in small glass-reactors (500 ml). Fermentation progress was monitored through the amount of CO_2_ released by a daily weighing measurement of glass-reactor to assess the weight loss. For both samples, from grapes and fermented vats, sampling was performed when fermentation reached about 2/3 of the sugar consumption or had been stopped. 166 grape fermentations and 11 fermented vats were showed to reach 2/3 of sugar consumption and then were sampled. Fermented musts were plated at different dilutions (10^–4^, 10^–5^ and 10^–6^) onto YPD (yeast extract, 1% w/v, peptone, 1% w/v, glucose, 2% w/v, agar 2% w/v) with 100 µg ml^−1^ of chloramphenicol and 150 µg ml^−1^ of biphenyl to delay bacterial and mold growth. At the optimal dilution, a maximum of 30 colonies were randomly collected after incubation (2 days at 26 °C) for a given sample, thus resulting in 3369 colonies. After two sub-cloning on YPD plates, each colony was stored in (30%, v/v) glycerol at − 80 °C. For the fermenting must samples from vats, the same dilutions were made and a maximum of 40 randomly chosen colonies were collected after incubation (2 days at 26 °C) for a given dilution. After two sub-clonings on YPD plates, each colony was stored in (30%, v/v) glycerol at − 80 °C.

In addition to the collected samples, 33 yeasts strains of diverse origins whose genome had recently been sequenced^49,59^, (Supplementary Table S3) and 35 commercial wine strains (Supplementary Table S4) widely used in Bordeaux wine estates were added to the collection. For 6 commercial strains among the most frequently used (522D, F33, FX10, F15, VL1, X5), we analyzed the genetic diversity of two batches of production of Active Dry Yeast (ADY), except for strain X5, for which only one batch was available. Four to ten single yeast cells were isolated with a Singer micromanipulator from each batch, thus resulting in 110 additional commercial wine yeast isolates into the collection.

### Molecular methods and genotyping

Yeast colonies of all grapes and vats samples were cultivated on differential WL nutrient agar medium (2 days at 26 °C) which generated a specific coloration depending on their genus and 2 of each type of colonies were filed on FTA cards for DNA transfer. The PCR amplification of the ITS region with primers ITS1 and ITS4^60^ was used to identify and select *Saccharomyces* colonies^61^. Each colony on WL medium corresponding to *Saccharomyces* was suspended in 20 µl of MilliQ water and analyzed by optical density at 660 nm. A readjustment of the amount of MilliQ water was made to obtain a final OD in the suspension cell of 10. All of these cell suspensions were then genotyped using 2 multiplex PCR reaction of 9 microsatellites loci (Supplementary Table S5^25,62–66^. The 2 multiplex PCR contained (for 8 samples) a total of 15.5 μl multiplexed primers, 50 µl of QIAGEN Multiplex PCR kit Master Mix and 18.5 μl water MilliQ. The PCRs were run in a final volume of 12 μl containing 2 μl of cell suspension. The following PCR program was used in the routine: initial denaturation at 95 °C for 15 min followed by 35 cycles of 95 °C for 30 s, 57 °C for 2 min, 72 °C for 1 min and finally a final extension at 60 °C for 30 min. PCR products were sized on a capillary electrophoresis ABI3730 (APPLIED BIOSYSTEMS) using size standard 600LIZ (GENESCAN). Locus YLL049W providing non-reproducible amplification was removed for the subsequent structure and diversity analyses. Capillary electrophoresis runs were read using GENE MARKER (V2.4.0,) and the sizes of microsatellites amplicons were recorded to investigate the genetic relationships between strains. The presence of missing values was allowed up to 3 loci per individual and these were taken into account in the analyses in order to obtain a comprehensive picture of yeast diversity.

### Data analysis

We calculated three diversity indices using ESTIMATES V9^67^: the Shannon (H′) index that measure the diversity within a population and take into account both richness and evenness, the Simpson index (D) with its opposite Simpson’s index of diversity (1 − D) which gives more weight to common or dominant species, and the Pielou evenness index (J′). Estimation of population diversity by rarefaction of 10,000 individuals was repeated 10 times. H′ was determined with the following equation:

${H}^{^{\prime}}=-{\sum }_{i=1}^{S}Pi.lnln \left(Pi\right)$, and D following the equation: $D={\sum }_{i=1}^{S}\frac{Ni\left(Ni-1\right)}{N\left(N-1\right)}$. With S the total number of genotypes in the population, the term Pi calculated as follows: $Pi=\frac{Ni}{N}$, Ni the number of individuals for genotype i and N the total number of unique genotypes. GENCLONE software (V2.0)^68^ was used to remove from our dataset strains with similar profiles resulting from potential clonal expansion. Strains profile comparison with yeast starters was performed using the BIONUMERICS V5.1 software (APPLIED MATHS, Belgium) with the categorical coefficients associated with the ward algorithm^69^ from the microsatellite data size. Strains sharing more than 75% of alleles at 17 loci with commercial strains yeast starters and no missing values were considered as related to these starters. They were retained for spanning tree drawing and removed from the different datasets. These spanning trees were drawn with the BIONUMERICS V5.1 software. Dendrograms were constructed using Bruvo’s distance^50^ as proposed by the POPPR 2.02^70^ and neighbor-joining clustering with ape 3.2^71^ under the R environment v3.5.2 (R DEVELOPMENT CORE TEAM 2011). The Bruvo distance requires perfect microsatellite loci, which is almost the case for 17 out of 18 loci but not for locus C4 which is composed of two motifs (one locus over 17). The use of this distance provides a phylogenic signal closer to genome sequencing than that observed with Dc Chord distance^9,72^. As a consequence, we have retained this distance, despite potential violation of the initial model proposed by Bruvo et al.^50^.

In order to assess the robustness of tree nodes, bootstrap resampling was performed by means of R and the pvclust 1.3-2 package^73^ and inferred with MEGA6, all bootstraps lower than 25 were not shown in the trees.

Population structure was evaluated using the Bayesian clustering method implemented by the software InStruct that considers the inbreeding is the main sexual mode of reproduction^51^ which is the case for yeast. Five chains of 150,000 iterations with a burn-in of 5000 were run for K = 1 to K = 25. The most likely number of ancestry’s populations was selected choosing the lowest DIC (Deviance information criterion). Bar plots presenting the ancestry profile for each population were drawn from the InStruct output file using an R script. On each population, basic statistics were estimated with poppr v2.8.3 and the DiveRsity v1.9.90 package, and provided as averages between loci. AMOVA pas performed with the Pegas package v0.12 as proposed by the poppR R package. Pairwise Fst distance and significance tests, and the Mantel test evaluating the correlation between geographic distance and genetic divergence, were performed as implemented with GenAlex v6.5^74–76^. Population geographic differentiation was also performed from the ancestry profile as implemented by the OBSTRUCT V1.0 software^52^.

Relative directional migration rates between the five appellations were estimated using the approach proposed by Sundqvist et al.^53^ with the website divMigrate (https://popgen.shinyapps.io/divMigrate-online/), using the D distance^77^. Directional migration rates between the four appellations, Medoc (38 genotypes), Pessac Leognan (89 genotypes), Saint-Emilion (7 genotypes) and Entre deux-Mers (5 genotypes), were inferred using Migrate 4.2.14, assuming constant population size^78^. The dataset included only isolates from the 2012 sampling year, and the 4 loci (YLR, SCAAT6, YKR072c and SCAAT2) which contained several missing data were removed. Identical clones were discarded after GENECLONE analysis, and isolates related to LSA were removed from this dataset.

For grape and cellar population comparison, data sets containing a maximum of 20 individuals were built by random sampling using a custom R script, from the data of the 4 cellars (or grapes) from Pessac Leognan, Bergerac and Saint Emilion in order to avoid unbalanced sampling between cellars and between regions. Fst were estimated from the sampled population using a custom R script and Fst calculated using the Hierfstat v0.04-22 package under the R environment. This randomized dataset was then further used for the estimation of exchanges between cellars and grapes using Migrate 4.2.14. Given the small proportion of missing data, the 17 loci were used for the analysis. All these datasets are available on the Open Data portal of INRAE (https://doi.org/10.15454/GMRGPO).

## Supplementary information

Supplementary Information.
