# Supplementary material for: Quantifying the effect of human practices on S. cerevisiae vineyard metapopulation diversity
Source: Sci Rep. 2020 Oct 1;10:16214. doi: 10.1038/s41598-020-73279-7 (PMC7530672; doi:10.1038/s41598-020-73279-7)
Supplement: Supplementary file 1 — Supplementary Information. [file 41598_2020_73279_MOESM1_ESM.docx]

**SUPPLEMENTARY INFORMATIONS**

Quantifying the effect of human practices on *S. cerevisiae* vineyard metapopulation diversity

Marine Börlin^a^, Olivier Claisse^a,f^,, Warren Albertin^a,b^, Franck Salin^c^, Jean-Luc Legras^d*^, Isabelle Masneuf-Pomarede^a,e*^


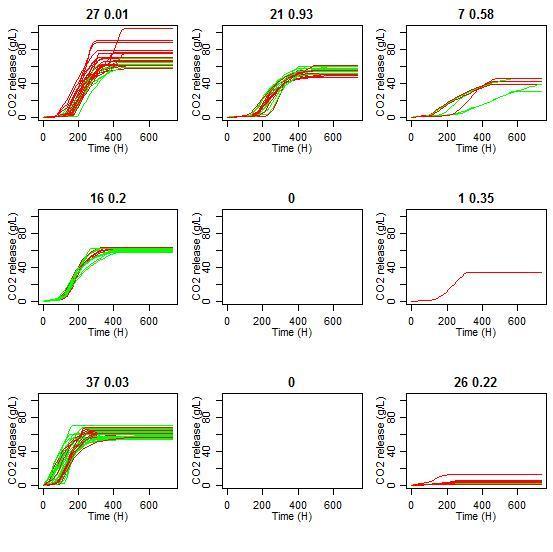

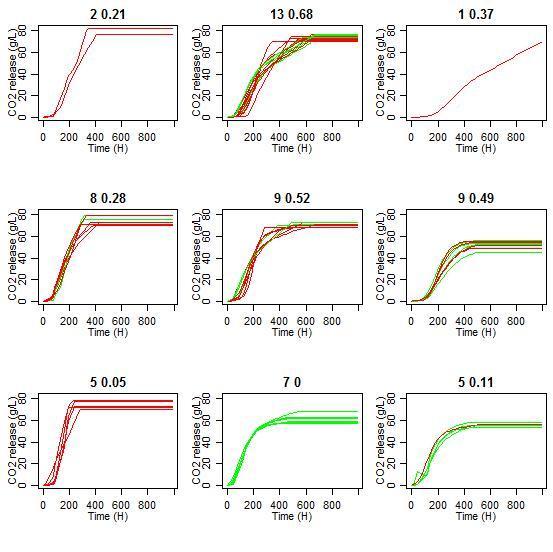


b.

a.

**Figure S1.**

Grape sample fermentation curves showing the consumption of CO_2_ in function of time in hours. **a.** 2012, 134 fermentations in which blue selection represent fermentation that did reach at least 50% of the alcoholic fermentation and in orange fermentation that stopped before the 50% of alcoholic fermentation. **b.** 2013, 59 fermentations that all did reach at least 50%. Curves clustered depending on the CO_2_ consumption on the vertical axis and the lag time on the horizontal axis. Samples from organic and conventional farming systems were in green and red, respectively.

**Figure S2.**
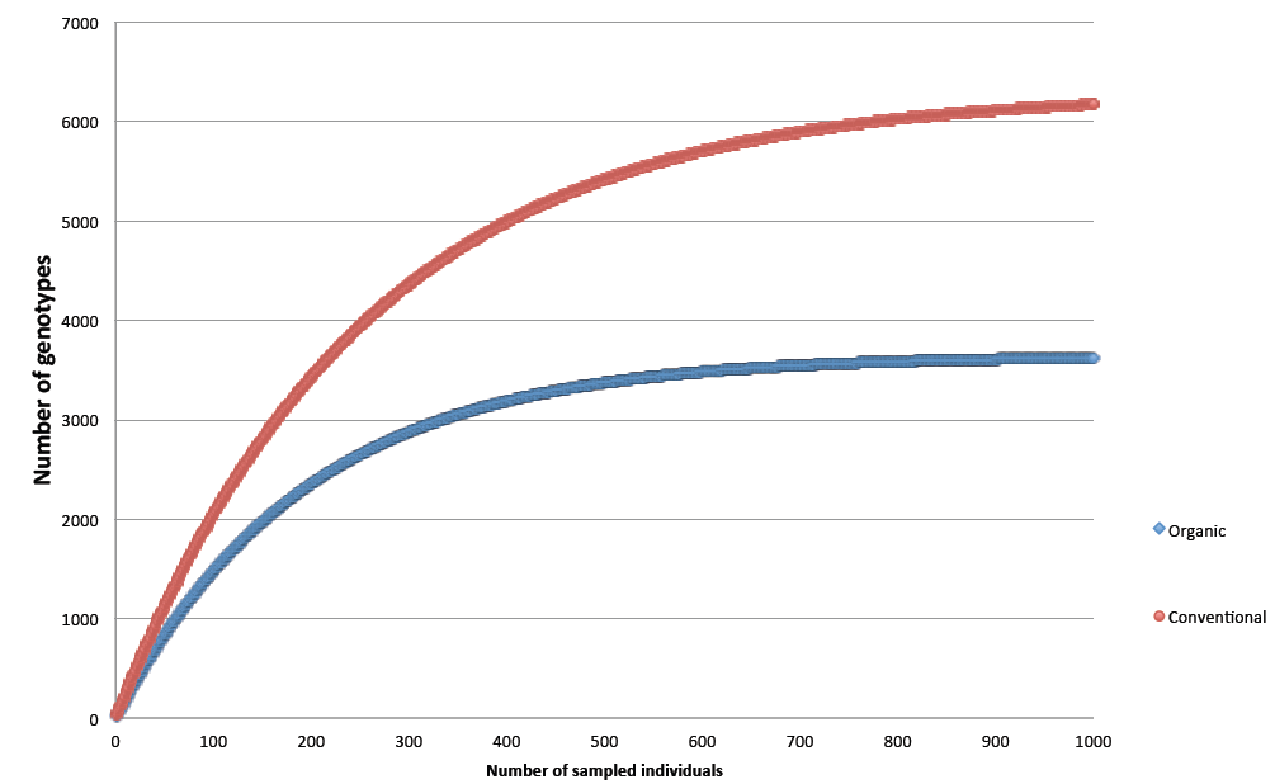


Comparison of genotypes rarefaction curves obtained for grapes sampled vineyards managed in organic (blue) and conventional (red) farming system. All calculations were performed using EstimateS v9.10.


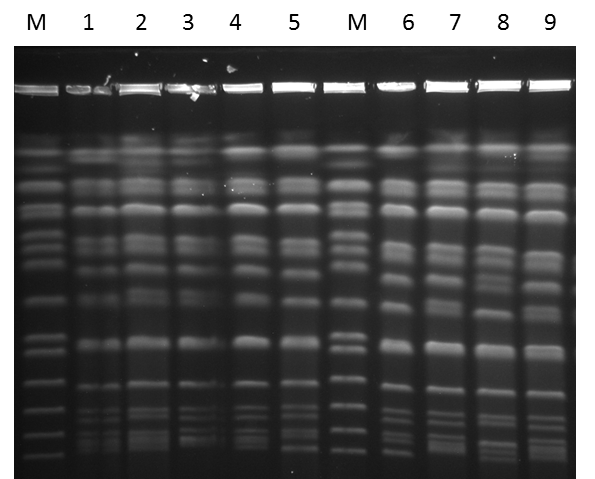


a.


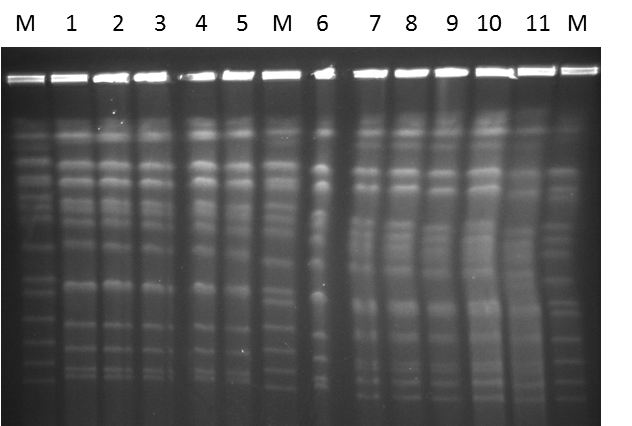


b.

**Figure S3.**

Example of karyotypes analysis of commercial strains and associated microsatellites patterns clonal variants isolated from the commercial lots and vineyards. a. M: molecular marker, 1-5: clonal variants of the commercial lot of F33, 6: clonal variants of the commercial lot of 522D; 7: 13caMconv4_3, 8: 13maMconv3_12, 9: 12yfMconv1_9. b. M: molecular marker, 1: F15, 2: 13hpMconv2_4, 3: 13caMconv4_9, 4: 12fz1Mconv5_11, 5: 12fz1Mconv5_24, 6: 12fz1Mconv5_29, 7:VL1, 8: 12bcMbio1_16, 9: 12bcMbio1_10, 10: 12bcMbio1_14, 11: 13maMbio1_10.

**Fig S4.**

Influence of the distance from the closest cellar to the grape sample site for each appellation a. on the percentage of grape fermentations that provided *S.cerevisiae* strains and b. on the percentage of commercial related grape strains found.

**A**

**B**

**Fig. S5:** Global evaluation of population structure with DAPC. Scatter plots presenting the contribution of different wine estate to the global diversity for axis 1 and 2 (A) and 2 and 3 (B).


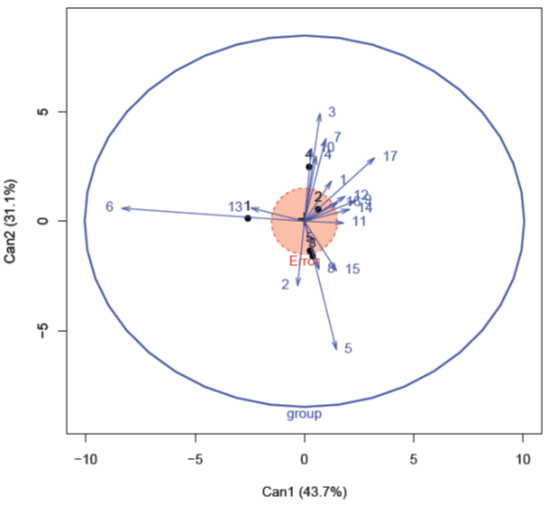


**Figure S6**. Canonical discriminant analysis on the *S. cerevisiae* dataset. The HE plot shows the relation of variation in the group means on two variables relative to the error variance. The arrows indicate the position of the inferred populations relative to the axes obtained by the canonical discriminant analysis. The black points indicate predefined populations (1 = Médoc; 2 = Pessac Léognan; 3 = Saint Emilion; 4 = Bergerac; 5 = Entre Deux-Mers) while numbers at the arrows indicate inferred populations.

**Figure S7. Relative migration network between the 5 appellations of the Bordeaux region. Significance is tested by 1000 permutations**

**Table S1.**

Assembly of the different site selected with the given code, the region, the farming system, the domains names, domain abbreviations, GPS data for cellars, site of sampling and distance between them. *Those samples were closer to others cellars than the one from the wine estate. The distances to a cellar were 350m for B1, 220m for B2 and 280m for E4.

| **Sub-region** | **Farming system** | **Name** | **Abbreviation** | **Cellar GPS data** | **Sampling GPS data** | **Distance (m)** |
| --- | --- | --- | --- | --- | --- | --- |
| Bergerac | Organic | Domaine Des Costes | cos | 44°51'33.0"N 0°30'35.0"E | - |  |
| Bergerac | Organic | Château Richard | ri | 44°46'45.4"N 0°18'56.3"E | 44°46'39.2"N 0°18'59.4"E | 197 |
| Medoc | Organic | Château Margaux | ma | 45°02'39.9''N 0°40'07.5''W | 45°02'47.2''N 0°41'10.1''W | 1380* |
| Medoc | Conventional | Château Margaux | ma | 45°02'39.9''N 0°40'07.5''W | 45°02'49.8''N 0°41'11.6''W | 1440* |
| Medoc | Organic | Château Palmer | pa | 45°02'12.2''N 0°40'10.8''W | 45°01'45.8''N 0°40'58.3''W | 1360* |
| Medoc | Conventional | Château Palmer | pa | 45°02'12.2''N 0°40'10.8''W | 45°01'46.3''N 0°40'58.2''W | 1350* |
| Medoc | Conventional | Château Camensac | cm | 45°08'47.8''N 0°47'11.4''W | 45°08'49.2"N 0°47'07.8"W | 81 |
| Pessac – Leognan | Organic | Château Baulos Charmes | bc | 44°44'09.3"N 0°32'26.3"W | 44°44'07.5"N 0°32'27.4"W | 66 |
| Pessac – Leognan | Conventional | Château Carbonnieux | ca | 44°44'41.9"N 0°34'07.9"W | 44°44'42.5"N 0°34'05.9"W | 54 |
| Pessac – Leognan | Conventional | Domaine De Chevalier | ch | 44°43'06.9"N 0°37'57.7"W | 44°43'02.2"N 0°38'06.7"W | 233 |
| Pessac – Leognan | Conventional | Château Fieuzal | fz | 44°42'50.8"N 0°36'23.5"W | 44°42'54.6"N 0°36'23.1"W | 124 |
| Pessac – Leognan | Organic | Château Bichon Cassignol | bi | 44°41'10.3"N 0°32'02.2"W | 44°41'17.3"N 0°32'15.0"W | 358 |
| Pessac – Leognan | Organic | INRA | in | 44°47'18.6"N 0°34'41.3"W | 44°47'23.0"N 0°34'43.2"W | 161 |
| Pessac – Leognan | Conventional | INRA | in | 44°47'18.6"N 0°34'41.3"W | 44°47'23.0"N 0°34'43.2"W | 161 |
| Pessac – Leognan | Conventional | Château Luchey Halde | lh | 44°49'12.5"N 0°37'49.5"W | 44°49'13.3"N 0°37'50.5"W | 38 |
| Entre Deux-Mers | Organic | Domaine Du Bourdieu | du | 44°41'54.4"N 0°16'23.2"W | 44°41'58.5"N 0°16'27.0"W | 152 |
| Entre Deux-Mers | Conventional | Château Ducourt | dc | 44°42'16.5"N 0°14'51.8"W | 44°42'09.9"N 0°14'41.3"W | 308 |
| Saint Emilion | Organic | Château Bellevue | be | 44°57'07.8"N 0°06'18.3"W | 44°57'15.0"N 0°06'12.9"W | 28 |
| Saint Emilion | Organic | Château Moulin de Lagnet | ml | 44°54'23.7"N 0°07'58.4"W | 44°54'24.3"N 0°07'57.4"W | 38 |
| Saint Emilion | Conventional | Château Haut Piquat | hp | 44°56'55.1"N 0°06'04.1"W | 44°56'57.0"N 0°06'15.4"W | 248 |
| Saint Emilion | Conventional | Couvent des Jacobins | cj | 44°53'40.8"N 0°09'18.6"W | 44°53'27.0"N 0°09'42.0"W | 661* |
| Saint Emilion | Conventional | Château Yon Figeac | yf | 44°54'17.8"N 0°10'59.7"W | 44°54'17.8"N 0°11'01.3"W | 44 |
| Saint Emilion | Organic | Château Gombard Guillot | gg | 44°55'55.1''N 0°12'28.7''W | 44°55'53"N 0°12'17"W | 263 |
| Saint Emilion | Organic | Château Beauséjour | beau | 44°55'28.4''N 0°04'33.6''W | 44°55'26"N 0°04'39"W | 140 |
| Saint Emilion | Conventional | Château Clinet | cli | 44°56'02.7''N 0°12'17.2''W | 44°55'56"N 0°12'13"W | 227 |
| Saint Emilion | Conventional | Château Soleil | so | 44°55'19.9''N 0°04'54.7''W | 44°55'26"N 0°04'39"W | 389 |
| Saint Emilion | Conventional | Montagne | mo | 44°57'27.2"N 0°10'23.1"W | - |  |

**Table S2.** Summary of grape and must samples collected in Bordeaux and Bergerac regions with indication of wine estate, sampling years, number of fermentations and number of *S.cerevisiae* profiles types *Also corresponding to the total number of fermentation for each domains **Sample with fermentations that ended between 28 and 100% of the alcoholic fermentation ***Number of distinct microsatellite profiles after clone removal with GenClone Nb: Number

|  |  | |  | | **2012** | | | | | | | | **2013** | | | | | | | **2012** | | | **2013** | | |
| --- | --- | --- | --- | --- | --- | --- | --- | --- | --- | --- | --- | --- | --- | --- | --- | --- | --- | --- | --- | --- | --- | --- | --- | --- | --- |
| **Appellation** | | **Wine estate** | | **Farming system** | | **Grape samples*** | **Nb of sample with fermentation**** | | **Nb. of fermentation giving S.c isolates** | | **Nb of *S.c* unique profiles***** | | **Grape samples*** | **Nb of sample with fermentation***** | | **Nb of fermentation giving S.c isolates** | | **Nb of *S.c* unique profiles***** | | **Must sample** | **Nb of *S.c* unique profiles***** | | **Must sample** | | **Nb of *S.c* unique profiles***** |
| Bergerac | | A1 | | Organic | | - | - | | - | | - | | - | - | | - | | - | | 1 | 32 | | - | | - |
| Bergerac | | A2 | | Organic | | 5 | 5 | | 3 | | 9 | | 5 | 5 | | 2 | | 6 | | 1 | 22 | | - | | - |
| Medoc | | B1 | | Organic | | 12 | 8 | | 1 | | 7 | | 6 | 6 | | 2 | | 12 | | - | - | | - | | - |
| Medoc | | B1 | | Conventional | | 3 | 1 | | 0 | | 0 | | 3 | 3 | | 2 | | 13 | | - | - | | - | | - |
| Medoc | | B2 | | Organic | | 3 | 2 | | 2 | | 12 | | - | - | | - | | - | | - | - | | - | | - |
| Medoc | | B2 | | Conventional | | 3 | 3 | | 1 | | 24 | | - | - | | - | | - | | - | - | | - | | - |
| Medoc | | B3 | | Conventional | | 5 | 4 | | 2 | | 7 | | - | - | | - | | - | | - | - | | - | | - |
| Pessac – Leognan | | C1 | | Organic | | 5 | 5 | | 2 | | 30 | | - | - | | - | | - | | 1 | 17 | | - | | - |
| Pessac – Leognan | | C2 | | Conventional | | 4 | 4 | | 2 | | 21 | | 5 | 5 | | 4 | | 46 | | - | - | | - | | - |
| Pessac – Leognan | | C3 | | Conventional | | 5 | 5 | | 0 | | 0 | | - | - | | - | | - | | - | - | | - | | - |
| Pessac – Leognan | | C4 | | Conventional | | 10 | 5 | | 3 | | 18 | | 5 | 5 | | 4 | | 13 | | - | - | | - | | - |
| Pessac – Leognan | | C5 | | Organic | | 7 | 7 | | 5 | | 26 | | 5 | 5 | |  | | 0 | | - | - | | - | | - |
| Pessac – Leognan | | C6 | | Organic | | 5 | 5 | | 1 | | 5 | | - | - | | - | | - | | - | - | | - | | - |
| Pessac – Leognan | | C6 | | Conventional | | 5 | 5 | | 2 | | 9 | | 6 | 6 | | 1 | | 4 | | - | - | | - | | - |
| Pessac – Leognan | | C7 | | Conventional | | 7 | 6 | | 5 | | 28 | | 4 | 4 | | 4 | | 18 | | - | - | | - | | - |
| Entre Deux-Mers | | D1 | | Organic | | 5 | 5 | | 1 | | 2 | | - | - | | - | | - | | 2 | 37 | | 1 | | 26 |
| Entre Deux-Mers | | D2 | | Conventional | | 5 | 3 | | 2 | | 5 | | - | - | | - | | - | | - | - | | - | | - |
| Saint Emilion | | E1 | | Organic | | 5 | 4 | | 0 | | 0 | | 5 | 5 | | 1 | | 6 | | 2 | 52 | | 1 | | 23 |
| Saint Emilion | | E2 | | Organic | | 5 | 5 | | 2 | | 2 | | 5 | 5 | |  | | 0 | | 1 | 22 | | - | | - |
| Saint Emilion | | E3 | | Conventional | | 5 | 1 | | 0 | | 0 | | 5 | 5 | | 4 | | 33 | | - | - | | - | | - |
| Saint Emilion | | E4 | | Conventional | | 5 | 5 | | 3 | | 8 | | 5 | 5 | | 2 | | 17 | | - | - | | - | | - |
| Saint Emilion | | E5 | | Conventional | | 4 | 4 | | 3 | | 12 | | - | - | | - | | - | | - | - | | - | | - |
| Saint Emilion | | E6 | | Organic | | 6 | 4 | | 1 | | 2 | | - | - | | - | | - | | - | - | | - | | - |
| Saint Emilion | | E7 | | Organic | | 5 | 4 | | 0 | | 0 | | - | - | | - | | - | | - | - | | - | | - |
| Saint Emilion | | E8 | | Conventional | | 5 | 2 | | 1 | | 7 | | - | - | | - | | - | | - | - | | - | | - |
| Saint Emilion | | E9 | | Conventional | | 5 | 5 | | 0 | | 0 | | - | - | | - | | - | | - | - | | - | | - |
| Saint Emilion | | E10 | | Conventional | | - | - | | - | | - | | - | - | | - | | - | | - | - | | 1 | | 12 |
| Total | | | | | | 134 | | 107 | | 42 | | 234 | 59 | | 59 | | 26 | | 168 | 8 | | 182 | 3 | 61 | |

**Table S3.**

33 *S.cerevisiae* strains of Sanger data base from wine and divers origins. OS column is the accession number in the internal collection at the University of Nottingham. OSNNN/A means a single spore was isolated from the original diploid and OSNNN/A/A indicates this process was repeated. The absence of any /A indicates that either the strain was haploid or that a monosporic culture was provided.

*Strains related to wine fermentation

| **OS** | **Strain** | **Geographic, Isolated by, Year and refereces** | **Source** |
| --- | --- | --- | --- |
| 288/A | 273614X | Royal Victoria Infirmary, Newcastle UK, Galloway A | Clinical isolate (Fecal) |
| 287/A | 378604X | Royal Victoria Infirmary, Newcastle UK, Galloway A | Clinical isolate (Sputum) |
| 181 | BC187* | Napa Valley, Bisson L, USA^Ref^ | Barrel Fermentation |
| 150/A | DBVPG1106* | Australia, 1947, Fornachon J | Grapes |
| 91/A | DBVPG1373 | Netherlands, Capriotti A, 1952^Ref^ | Soil |
| 84/A | DBVPG1788 | Turku, Finland, Capriotti A, 1957^Ref^ | Soil |
| 92/A | DBVPG1853 | Ethiopia, Rossi, 1959^Ref^ | White Teff |
| 155/A | DBVPG6040 | Netherlands, 1947^Ref^ | Fermenting fruit juice |
| 60/A | DBVPG6044 | West Africa, Guillermond A, 1925^Ref^ | Bili wine, from *Osbeckia grandiflora* |
| 3/A | DBVPG6765 | Unknown^Ref^ | Unknown |
| 251/A/A | K11 | Japan, 1981^Ref^ | Shochu sake strain |
| 220/A | L-1374* | Cauquenes, Chile, Ganga A, 1999 | Fermentation from must País |
| 21/A | L-1528* | Cauquenes, Chile, Ganga A, 1999 | Fermentation from must Cabernet |
| 247/A | NCYC110 | West Africa, Guillermond A, pre-1914^Ref^ | Ginger beer from *Z.officinale* |
| 96 | S288c | Merced, California, USA, Mrak E, 1938^Ref^ | Rotting fig |
| 17/A | SK1 | USA, Kane S, pre-1974^Ref^ | Soil |
| 278/A | UWOPS03-461.4 | Telok Senangin, Malaysia, Wiens F, 2003^Ref^ | Nectar, Bertram palm |
| 280/A | UWOPS05-227.2 | Telok Senangin, Malaysia, Lachance M, 2005 | *Trigona spp* (Stringless bee) collected near Bertam palm flower |
| 279/A | UWOPS05-217.3 | Telok Senangin, Malaysia, Lachance M, 2005 | Nectar, Bertram palm |
| 270/A | UWOPS83-787.3 | Great Inagua Island, Bahamas, 1983, Lachance M | Fruit, *Opuntia stricta* |
| 271/A | UWOPS87-2421 | Puhelu Road, Maui, Hawaii, Lachance M, 1987 | Cladode, *Opuntia megacantha* |
| 281 | W303 | Created by Rothstein R by multiple crossing^Ref^ | NA |
| 253/A/A | Y12 | Ivory Cost, pre-1981^Ref^ | Palm wine strain |
| 97/A | Y55* | France, Winge Ö, between 1930-60^Ref^ | Grape |
| 252/A | Y9 | Indonesia, pre-1962^Ref^ | Ragi (similar to sake wine) |
| 174 | YIIc17_E5* | Sauternes, France | Wine |
| 308/A | YJM975* | Ospedali Riuniti di Bergamo, Italy, 1994-6^Ref^ | Isolated from vagina of patient suffering from vaginitis |
| 303/A | YJM978* | Ospedali Riuniti di Bergamo, Italy, 1994-6^Ref^ | Isolated from vagina of patient suffering from vaginitis |
| 304/A | YJM981* | Ospedali Riuniti di Bergamo, Italy, 1994-6^Ref^ | Isolated from vagina of patient suffering from vaginitis |
| 182 | YPS606 | Pennsylvania, USA, Sniegowski P, 1999^Ref^ | Bark of *Q.rubra* |
| 258/A/A | YS2 | Australia^Ref^ | Baker strain |
| 259/A/A | YS4 | Netherlands, 1975, Barnett J^Ref^ | Baker strain |
| 262/A/A | YS9 | Singapore^Ref^ | Baker strain |

**Table S4.**

33 *S.cerevisiae* strains from industrial yeast production commonly used in Aquitaine region, in organic and conventional farming.

| **Commercial name** | **Strain** | **Distributor** |
| --- | --- | --- |
| Actiflore cerevisiae | 522D | Laffort |
| Excellence B2 |  | Lamothe Abiet |
| Lalvin BM 45 |  | Lallemand |
| Levuline CHP | CIVC8130 | Oenofrance |
| Lalvin CY 3079 | BourgoBlanc | Lallemand |
| Excellence C1 Val oeno |  | Lamothe Abiet |
| Zymaflore F10 | FZ 182 | Laffort |
| Zymaflore F15 |  | Laffort |
| Actiflore F33 | F33 | Laffort |
| Fermol Arome plus | PB2010 | Spindal |
| Fermivin | 7013 | Littorale |
| Zymaflore FX10 |  | Laffort |
| ICV-GRE | 138 grenache | ICV |
| K1 | Killer non marquée | Lallemand |
| L.A. L13 | L13 | Lamothe Abiet |
| Rhône L2226 |  | Lallemand |
| Lallferm bio |  | IOC / Lallemand |
| Lalvin QA23 | QA23 | Lallemand |
| Lalvin 71B | 71B | Lallemand |
| Levuline ALS | EG8 | Oenofrance |
| Levuline BRG | UP 30Y5 | Oenofrance |
| Oenoferm Bio |  | Littorale |
| Vitilevure KD | R2 | Martin Vialatte |
| Zymaflore RX60 | rx60 | Laffort |
| SP organic |  | Martin Vialatte |
| Uvaferm CEG | CEG - Epernay 2 | Lallemand |
| Achor vin 13 | vin 13 | Littorale |
| Vitilevure quartz |  | Martin Vialatte |
| Zymaflore VL1 | vl1 | Laffort |
| Zymaflore VL3 | lv3 | Laffort |
| Zymaflore X16 | x16 | Laffort |
| Zymaflore X5 | x5 | Laffort |
| Zymaflore 011 organiq |  | Laffort |

**Table S5.**

Microsatellite loci for *Saccharomyces cerevisiae* genotyping with, repeated motif, ORF, primer sequence, fluorescence dye, mix number and concentration used for 8 samples PCR mix preparation for each marker

| **Mix** | **Site name** | **Motif and type** | **ORF or coordinates** | **Primers** | **Fluorescent dye** | **Multiplex** | **Quantity (nM)** | **Author** |
| --- | --- | --- | --- | --- | --- | --- | --- | --- |
|  | ScAAT2 | TAA | YBL084c | FW: CAGTCTTATTGCCTTGAACGA | PET | 1 | 100 | 4 |
|  |  |  |  | RV: GTCTCCATCCTCCAAACAGCC |  |  |  |  |
|  | ScAAT3 | TAA | YDR160w | FW: TGGGAGGAGGGAAATGGACAG | NED | 1 | 200 | 1, 3 |
|  |  |  |  | RV: TTCAGTTACCCGCACAATCTA |  |  |  |  |
|  | C5 | GT | VI-210250/210414 | FW: TGACACAATAGCAATGGCCTTCA | VIC | 1 | 50 | 5 |
|  |  |  |  | RV: GCAAGCGACTAGAACAACAATCACA |  |  |  |  |
| **Mix1** | C3 | CAA | YGL139w | FW: CTTTTTATTTACGAGCGGGCCAT | NED | 1 | 100 | 5 |
|  |  |  |  | RV: AAATCTCATGCCTGTGAGGGGTAT |  |  |  |  |
|  | C8 | TAA | YGL014w | FW: CAGGTCGTTCTAACGTTGGTAAAATG | 6FAM | 1 | 25 | 5 |
|  |  |  |  | RV: GCTGTTGCTGTTGGTAGCATTACTGT |  |  |  |  |
|  | C11 | GT | X-518870/519072 | FW: TTCCATCATAACCGTCTGGGATT | 6FAM | 1 | 50 | 5 |
|  |  |  |  | RV: TGCCTTTTTCTTAGATGGGCTTTC |  |  |  |  |
|  | YKR072c | GAC | YKR072c | FW: AGATACAGAAGATAAGAACGAAAA | PET | 1 | 50 | 1, 2 |
|  |  |  |  | RV: TTATTGATGCTTATCTATTATACC |  |  |  |  |
|  | ScAAT6 | TAA | IX-105711/105883 | FW: TTACCCCTCTGAATGAAAACG | PET | 1 | 100 | 1, 3 |
|  |  |  |  | RV: AGGTAGTTTAGGAAGTGAGGC |  |  |  |  |
|  | SCYOR267c | TGT | YOR267c | FW: TACTAACGTCAACACTGCTGCCAA | VIC | 1 | 100 | 1, 4 |
|  |  |  |  | RV: GGATCTACTTGCAGTATACGGG |  |  |  |  |
|  | YKL172w | GAA | YKL172w | FW: CAGGACGCTACCGAAGCTCAAAAG | 6FAM | 2 | 25 | 2 |
|  |  |  |  | RV: ACTTTTGGCCAATTTCTCAAGAT |  |  |  |  |
|  | ScAAT1 | TTA | XIII-86902/87140 | FW: AAAGCGTAAGCAATGGTGTAGATACTT | VIC | 2 | 100 | 1, 3, 4 |
|  |  |  |  | RV: CAAGCCTCTTCAAGCATGACCTTT |  |  |  |  |
|  | C4 | TAA+TAG | XV-110701/110935 | FW: AGGAGAAAAATGCTGTTTATTCTGACC | NED | 2 | 200 | 5 |
|  |  |  |  | RV: TTTTCCTCCGGGACGTGAAATA |  |  |  |  |
| **Mix 2** | C9 | TAA | YOR156c | FW: AAGGGTTCGTAAACATATAACTGGCA | NED | 2 | 100 | 5 |
|  |  |  |  | RV: TATAAGGGAAAAGAGCACGATGGC |  |  |  |  |
|  | ScAAT5 | TAA | XVI-897051/8970210 | FW: AGCATAATTGGAGGCAGTAAAGCA | NED | 2 | 100 | 5 |
|  |  |  |  | RV: TCTCCGTCTTTTTTGTACTGCGTG |  |  |  |  |
|  | C6 | CA | XVI-485898/485996 | FW: GTGGCATCATATCTGTCAATTTTATCAC | VIC | 2 | 50 | 5 |
|  |  |  |  | RV: CAATCAAGCAAAAGATCGGCCT |  |  |  |  |
|  | YPL009c | CTT | YPL009c | FW: AACCCATTGACCTCGTTACTATCGT | 6FAM | 2 | 50 | 1, 4 |
|  |  |  |  | RV: TTCGATGGCTCTGATAACTCCATTC |  |  |  |  |
|  | YLR | TC | XII-823393/823562 | FW: CTGGAATGAAATTAAACAAAAGC | PET | 2 | 100 | 2 |
|  |  |  |  | RV: TCTTCCTTTTCTACTATCTTCTC |  |  |  |  |
|  | YLL049W | TA | XII-40666/41205 | FW: GCAACATAATGATTTTGAGGT | PET | 2 | 50 | 6 |
|  |  |  |  | RV: GTGTCTTGTGTGAGCATAGTGGAGAA |  |  |  |  |

Authors: (1) Field and Wills (1998), (2) Hennequin et al. (2001), (3) Perez et al. (2001), (4) Gonzalez Techera et al. (2001), (5) Legras et al. (2005), (6) Bradbury et al. (2006)

**Table S6.** Basic statistics about the sampling (A) and Locus based statistics . A-Basic statistics about the different population sampled after the removal of clones

| **Code Estate** | Vine  management | Num of individuals | Expected Num of multilocus genotypes | Shannon H | Simpson’s Index | Evenness (Pielou) | Allelic Richness | Obs  Het. | Nei’s gene diversity Hexp | Index asso Ia | | Fis | |
| --- | --- | --- | --- | --- | --- | --- | --- | --- | --- | --- | --- | --- | --- |
| cm | Conventionnal | 4 | 4 | 1.39 | 0.75 | 1 | 1.58 | 0.029 | 0.324 | 1.62 | 0.91 | |  |
| ma | Organic | 10 | 10 | 2.30 | 0.90 | 1 | 2.14 | 0.559 | 0.602 | 8.25 | 0.04 | |  |
| ma | Conventionnal | 1 | 1 |  |  |  |  |  |  |  |  | |  |
| pa | Organic | 12 | 10 | 2.49 | 0.92 | 1 | 1.58 | 0.059 | 0.356 | 3.90 | 0.86 | |  |
| pa | Conventionnal | 24 | 10 | 3.18 | 0.96 | 1 | 1.68 | 0.049 | 0.368 | 1.84 | 0.88 | |  |
| bc | Organic | 17 | 10 | 2.83 | 0.94 | 1 | 1.89 | 0.465 | 0.513 | 2.07 | 0.10 | |  |
| bi | Organic | 19 | 10 | 2.94 | 0.95 | 1 | 2.23 | 0.327 | 0.633 | 4.96 | 0.44 | |  |
| ca | Conventionnal | 51 | 9.96 | 3.91 | 0.98 | 0.989 | 1.91 | 0.16 | 0.504 | 1.13 | 0.66 | |  |
| fz | Conventionnal | 21 | 10 | 3.05 | 0.95 | 1 | 2.51 | 0.35 | 0.726 | 2.57 | 0.52 | |  |
| in | Organic | 6 | 6 | 1.79 | 0.83 | 1 | 1.49 | 0.116 | 0.326 | 12.03 | 0.66 | |  |
| in | Conventionnal | 9 | 9 | 2.20 | 0.89 | 1 | 2.21 | 0.319 | 0.617 | 5.21 | 0.47 | |  |
| lh | Conventionnal | 41 | 10 | 3.71 | 0.98 | 1 | 1.93 | 0.087 | 0.528 | 2.21 | 0.83 | |  |
| cj | Conventionnal | 22 | 10 | 3.09 | 0.96 | 1 | 2.05 | 0.332 | 0.551 | 3.08 | 0.43 | |  |
| cli | Conventionnal | 7 | 7 | 1.95 | 0.86 | 1 | 1.11 | 0 | 0.096 | 0.43 | 1 | |  |
| gg | Organic | 2 | 2 | 0.69 | 0.50 | 1 | 1.04 | 0.062 | 0.083 | NA | 0 | |  |
| ml | Organic | 2 | 2 | 0.69 | 0.50 | 1 | 1.77 | 0.206 | 0.422 | NA | 0.30 | |  |
| yf | Conventionnal | 9 | 9 | 2.20 | 0.89 | 1 | 1.93 | 0.403 | 0.520 | 5.88 | 0.25 | |  |
| be | Organic | 2 | 2 | 0.69 | 0.50 | 1 | 1.29 | 0.344 | 0.240 | NA | -0.89 | |  |
| hp | Conventionnal | 27 | 10 | 3.30 | 0.96 | 1 | 1.91 | 0.247 | 0.508 | 3.37 | 0.50 | |  |
| ri | Organic | 11 | 10 | 2.40 | 0.91 | 1 | 2.23 | 0.416 | 0.635 | 5.50 | 0.34 | |  |
| dc | Conventionnal | 4 | 4 | 1.39 | 0.750 | 1 | 1.642 | 0.031 | 0.413 | 7.22 | 0.94 | |  |
| du | Organic | 1 | 1 |  |  |  |  |  |  |  |  | |  |

B-Locus based statistics**.**

| Locus | Heterozygosity with population structure Hs | Heterozygosity without population structure Ht | Nei Gst | Gprimest | D (Jost 2008) |
| --- | --- | --- | --- | --- | --- |
| C11s | 0.586 | 0.903 | 0.352 | 0.874 | 0.802 |
| C3s | 0.575 | 0.734 | 0.217 | 0.529 | 0.393 |
| C4s | 0.511 | 0.850 | 0.399 | 0.839 | 0.727 |
| C5s | 0.596 | 0.848 | 0.297 | 0.760 | 0.654 |
| C6s | 0.510 | 0.718 | 0.290 | 0.612 | 0.445 |
| C8s | 0.617 | 0.818 | 0.246 | 0.665 | 0.550 |
| C9s | 0.221 | 0.337 | 0.345 | 0.456 | 0.156 |
| ScAAT1s | 0.576 | 0.902 | 0.362 | 0.878 | 0.806 |
| ScAAT2s | 0.422 | 0.662 | 0.362 | 0.645 | 0.434 |
| ScAAT3s | 0.573 | 0.828 | 0.308 | 0.745 | 0.626 |
| ScAAT5s | 0.397 | 0.666 | 0.404 | 0.689 | 0.468 |
| ScAAT6s | 0.397 | 0.525 | 0.244 | 0.418 | 0.222 |
| SCYOR267Cs | 0.560 | 0.858 | 0.348 | 0.814 | 0.710 |
| YKL172Ws | 0.214 | 0.425 | 0.496 | 0.646 | 0.281 |
| YKR072Cs | 0.151 | 0.392 | 0.614 | 0.736 | 0.297 |
| YLRs | 0.566 | 0.813 | 0.304 | 0.734 | 0.610 |
| YPL009Cs | 0.479 | 0.775 | 0.383 | 0.755 | 0.596 |
| global | 0.468 | 0.709 | 0.341 | 0.659 | 0.419 |

**Table S7.**

Pulsed field electrophoresis profiles obtained for the commercial strains and the associated microsatellites patterns clonal variants isolated either from the commercial lots or vineyards. In each group of strain, the commercial strains of reference are the first of the group and is indicated in bold.

*Strains used for diversity and populations structure analysis

| **Strains** | **Wine estate** | **Profiles ECP** |
| --- | --- | --- |
| **FX10** |  | I |
| 13caMconv3_9 | C2 | I |
| 13lhMconv3_10 | C7 | I |
| 13lhMconv3_15 | C8 | I |
| 13lhMconv3_22 | C9 | I |
| 13lhMconv2_26 | C10 | I |
| 12caMconv3_12 | C2 | II |
| 13beMbio3_10 | E1 | I |
| 12cmMconv5_10 | B3 | I |
| **X5** |  | III |
| 12piMbio3_1 |  | III |
| 12piMbio3_9 |  | III |
| 12riMbio2_28 |  | III |
| 12riMbio3_24 |  | III |
| **F15** |  | IV |
| 13hpMconv2_4 | E3 | IV |
| 13caMconv4_9 | C2 | IV |
| 12fz1Mconv5_11 | C4 | IV |
| 12fz1Mconv5_24 | C4 | IV |
| 12fz1Mconv5_29 | C4 | IV |
| **VL1** |  | V |
| 12bcMbio1_16 | C1 | V |
| 12bcMbio1_10 | C1 | V |
| 12bcMbio1_14 | C1 | V |
| 13maMbio1_10 | B1 | V |
| **F33_16_1*** |  | VI |
| **F33_16_7** |  | VI |
| **F33_16_8** |  | VI' |
| **F33_18_2** |  | VI'' |
| **F33_18_5** |  | VI |
| **F33_18_8** |  | VI |
| **F33_18_10** |  | VI''' |
| **Acti522D*** |  | VII |
| **522_16_1** |  | VII' |
| **522_17_6** |  | VI''' |
| **522_17_8** |  | VI''' |
| 13caMconv4_3 | C2 | VI'' |
| 13maMconv3_12 | B1 | VI'''' |
| 12yfMconv1_9 | E5 | VI'' |
| 12yfMconv1_9 | E5 | VI'' |

**Table S8.**

Median directional estimates of migration rate (Nm) as calculated by MIGRATE with 95% confidence intervals (median estimate: lower 95% confidence interval – upper 95% confidence interval). Appellations code: 1 – Médoc, 2 – Pessac Léognan, 3 – Saint Emilion, 4 – Entre Deux Mers.

| **Parameter** | **Nm** | **Lower confidence value** | **Higher confidence value** |
| --- | --- | --- | --- |
| M_2->1 | 6.8 | 2.7 | 11.2 |
| M_1->2 | 2.2 | 0.0 | 4.1 |
| M_3->1 | 9.8 | 5.2 | 14.3 |
| M_1->3 | 4.5 | 1.1 | 7.9 |
| M_3->2 | 7.8 | 3.1 | 12.3 |
| M_2->3 | 8.8 | 5.5 | 11.8 |
| M_4->1 | 9.8 | 4.8 | 15.3 |
| M_1->4 | 4.3 | 1.5 | 6.7 |
| M_2->4 | 10.4 | 5.2 | 16.7 |
| M_4->2 | 6.0 | 2.5 | 9.5 |
| M_4->3 | 11.4 | 6.9 | 15.9 |
| M_3->4 | 11.5 | 7.1 | 15.7 |

**Table S9.**

Correlation between the ancestry profile of sampled population as estimated with Obstruct (Gayevskiy et al. 2014)**.** Pairwise matrix of R^2^ values between the 5 appellations of Bordeaux and Bergerac regions, after removing all related to commercial grape strains. The significance of the correlation between is tested by 10000 permutations. P-values are given between brackets.

|  | Pessac Léognan | Entre Deux-Mers | Médoc | Bergerac | Saint Emilion |
| --- | --- | --- | --- | --- | --- |
| Pessac Léognan | **NA** | 0.02 (0.02) | 0.08 (<0.001) | 0.02 (<0.001) | 0.06 (<0.001) |
| Entre Deux-Mers | 0.02 (0.02) | **NA** | 0.07 (0.01) | 0.23 (0.02) | 0.04 (0.16) |
| Médoc | 0.08 (<0.001) | 0.07 (0.01) | **NA** | 0.10 (<0.001) | 0.12 (<0.001) |
| Bergerac | 0.02 (<0.001) | 0.23 (0.02) | 0.10 (<0.001) | **NA** | 0.08 (<0.001) |
| Saint Emilion | 0.06 (<0.001) | 0.04 (0.16) | 0.12 (<0.001) | 0.08 (<0.001) | **NA** |

**Table S10. Estimations of directional migration rates with divMigrate (A) and Migrate (B)**

A- Relative directional migration estimates of migration rate as calculated by div-Migrate. Significance of the differences between the two directional migration estimates is obtained by 1000 bootstrap resampling and indicated by *.

| Appellation | Medoc | Pessac Leognan | Saint Emilion | Bergerac | Entre 2 Mer |
| --- | --- | --- | --- | --- | --- |
| Medoc | NA | 0.149 | 0.099 | 0.06 | 0.072 |
| Pessac Leognan | 0.722* | NA | 0.529 | 0.184 | 0.185 |
| Saint Emilion | 0.851* | 1* | NA | 0.13 | 0.21 |
| Bergerac | 0.2 | 0.3 | 0.203 | NA | 0.125 |
| Entre 2 Mer | 0.227 | 0.303 | 0.474 | 0.097 | NA |

B- Mean directional estimates of migration rate (Nm) as calculated by MIGRATE with 95% confidence intervals (mean estimate: lower 95% confidence interval – upper 95% confidence interval). All estimation goes from the appellation on the left to the appellation of the top line.

| Appellation | Médoc | Pessac Léognan | Saint Emilion | Entre Deux-Mers |
| --- | --- | --- | --- | --- |
| Médoc |  | 2.0: 0-4 | 4.5: 1.1-8.0 | 4.4: 1.5-6.7 |
| Pessac Léognan | 6.9: 2.7-11.2 |  | 8.8:5.53-11.8 | 10.7: 5.2-16.7 |
| Saint Emilion | 10.0:5.2-14.33 | 7.8:3.1-12.3 |  | 11.6: 7.1-15.7 |
| Entre Deux-Mers | 9.9: 4.8-15.3 | 6.0: 2.5-9.5 | 11.5: 6.9-15.9 |  |

**Table S11.** **Differentiation** between wine estates and grapes populations, as measured from pairwise Fst statistics. Fst have been estimated between a. cellar and grape population of the same appellation and b. between cellar population and grape from the whole Aquitaine region. Significance of differentiation were estimated by comparison with 10000 permutations.

a

| **Cellars** | cos | ri | bc | du | be | ml | mo |
| --- | --- | --- | --- | --- | --- | --- | --- |
| **Number of grape strains** | 11 | 11 | 164 | 5 | 71 | 71 | 71 |
| **Number of cellar strains** | 28 | 20 | 12 | 61 | 75 | 20 | 9 |
| **Fst** | 0.215 | 0.136 | 0.214 | 0.158 | 0.129 | 0.087 | 0.092 |
| **p-value** | 0.001 | 0.001 | 0.001 | 0.001 | 0.001 | 0.001 | 0.001 |

b

| **Cellars** | cos | ri | bc | du | be | ml | mo |
| --- | --- | --- | --- | --- | --- | --- | --- |
| **Number of grape strains** | 302 | 302 | 302 | 302 | 302 | 302 | 302 |
| **Number of cellar strains** | 28 | 20 | 12 | 61 | 75 | 20 | 9 |
| **Fst** | 0.133 | 0.067 | 0.201 | 0.057 | 0.109 | 0.042 | 0.04 |
| **P-value** | 0.001 | 0.001 | 0.001 | 0.001 | 0.001 | 0.001 | 0.002 |
